# Evaluating the Accuracy of Large Language Model (ChatGPT) in Providing Information on Metastatic Breast Cancer

**DOI:** 10.34172/apb.2024.060

**Published:** 2024-07-31

**Authors:** Ramakrishna Gummadi, Nagasen Dasari, D. Sathis Kumar, Sai Kiran S.S. Pindiprolu

**Affiliations:** Aditya Pharmacy College, Surampalem, Andhra Pradesh, 533 437, India.

**Keywords:** Artificial intelligence, ChatGPT, Breast cancer, Patient education, Healthcare

## Abstract

**Purpose:**

Artificial intelligence (AI), particularly large language models like ChatGPT developed by OpenAI, has demonstrated potential in various domains, including medicine. While ChatGPT has shown the capability to pass rigorous exams like the United States Medical Licensing Examination (USMLE) Step 1, its proficiency in addressing breast cancer-related inquiries—a complex and prevalent disease—remains underexplored. This study aims to assess the accuracy and comprehensiveness of ChatGPT’s responses to common breast cancer questions, addressing a critical gap in the literature and evaluating its potential in enhancing patient education and support in breast cancer management.

**Methods:**

A curated list of 100 frequently asked breast cancer questions was compiled from Cancer.net, the National Breast Cancer Foundation, and clinical practice. These questions were input into ChatGPT, and the responses were evaluated for accuracy by two primary experts using a four-point scale. Discrepancies in scoring were resolved through additional expert review.

**Results:**

Of the 100 responses, 5 were entirely inaccurate, 22 partially accurate, 42 accurate but lacking comprehensiveness, and 31 highly accurate. The majority of the responses were found to be at least partially accurate, demonstrating ChatGPT’s potential in providing reliable information on breast cancer.

**Conclusion:**

ChatGPT shows promise as a supplementary tool for patient education on breast cancer. While generally accurate, the presence of inaccuracies underscores the need for professional oversight. The study advocates for integrating AI tools like ChatGPT in healthcare settings to support patient-provider interactions and health education, emphasizing the importance of regular updates to reflect the latest research and clinical guidelines.

## Introduction

 Artificial Intelligence (AI) has rapidly emerged as a transformative force across various domains, particularly in the field of medicine.^1^ Among these innovations, ChatGPT, a next-generation large language model developed by OpenAI, stands out for its proficiency in generating human-like responses to diverse user inquiries on a wide array of subjects. Since its launch in November 2022, ChatGPT has garnered immense popularity, amassing over 100 million users within a mere two months and generating a staggering 1.5 billion visits per month.^2^

 ChatGPT’s potential in revolutionizing medical practice is particularly noteworthy. It achieved a passing score on the United States Medical Licensing Examination (USMLE) Step 1, demonstrating its capability in medical knowledge.^3,4^ Moreover, in comparative assessments of responses to patient queries, ChatGPT’s answers were rated higher in quality and empathy than those provided by physicians.^5,6^ These advancements highlight the growing interest in integrating ChatGPT into healthcare.

 However, the integration of ChatGPT and similar LLMs into healthcare also brings certain challenges and potential negative effects. On the positive side, ChatGPT can enhance accessibility to medical information, provide timely responses, and support patient education.^7^ It can serve as a valuable resource for preliminary information gathering and improve patient engagement. Conversely, there are concerns about the accuracy and reliability of the information provided by ChatGPT, as it may generate responses that are partially accurate or entirely inaccurate.^8^ The static nature of its knowledge base means it cannot incorporate the most recent research or clinical guidelines unless periodically updated. Furthermore, the use of AI in healthcare raises ethical considerations, such as patient privacy and the potential for over-reliance on AI tools at the expense of professional medical advice.^9^

 Recent investigations have illuminated ChatGPT’s accuracy and utility in addressing specialty-specific inquiries across various medical disciplines, including bariatric surgery, cirrhosis and hepatocellular carcinoma, and cardiovascular disease.^9-11^ Despite these promising developments, there remains a critical gap in the literature concerning ChatGPT’s proficiency in responding to inquiries related to breast cancer, a significant and prevalent oncological condition affecting millions worldwide.^12^

 Breast cancer represents a complex disease spectrum characterized by diverse manifestations, ranging from prevention strategies to diagnosis, treatment modalities, and survivorship considerations.^13,14^ Given the multifaceted nature of breast cancer and the profound impact it exerts on patients’ lives, accurate and empathetic information dissemination is paramount.^15-17^ Thus, evaluating ChatGPT’s performance in generating responses to commonly asked questions about breast cancer holds immense clinical and research significance.

 In light of the notable successes achieved by ChatGPT in other medical domains, it is imperative to investigate its efficacy in addressing inquiries specific to breast cancer. Such an evaluation not only holds the potential to enhance patient education and support but also provides valuable insights into the capabilities and limitations of AI-driven healthcare solutions. Therefore, this study aims to fill this critical gap by systematically assessing ChatGPT’s proficiency in generating accurate and comprehensive responses to commonly asked questions pertaining to breast cancer. Through this work, we aim to contribute to the growing body of literature on AI applications in healthcare and inform future developments aimed at optimizing patient care in the realm of breast cancer management.^18^

## Methodology

###  Questions curation and source of data

 A list of 100 questions for entry into the ChatGPT (Verson 4.0) user interface were curated. from frequently asked questions listed on Cancer.net and the National Breast Cancer Foundation’s website at https://www.nationalbreastcancer.org/breast-cancer-faqs, combined with inquiries commonly received in their clinical practice. These questions were carefully chosen to reflect the real-world concerns and informational needs of patients regarding breast cancer.^18^

## ChatGPT

 ChatGPT, developed by OpenAI, is a sophisticated language model trained on a vast array of data sources, including websites, books, and articles available up until early 2021. This extensive training dataset enables ChatGPT to generate articulate, conversational, and comprehensible replies to a wide variety of queries. To enhance its performance and ensure it adheres to user instructions accurately, the model underwent fine-tuning through a process called Reinforcement Learning from Human Feedback (RLHF). In this process, human evaluators provided feedback that acted as a reward signal, allowing the model to learn and adapt to a broad range of commands and written instructions based on human preferences. Moreover, efforts were made to align the model’s responses with user intentions while actively working to reduce biases and the likelihood of generating toxic or harmful content. The precise data sources utilized for ChatGPT’s training are not publicly disclosed, ensuring a broad and diverse foundation for its knowledge and capabilities.^19-23^

## Categories and scoring criteria for responses

 The collected questions were organized into four thematic categories: diagnosis (17 questions), treatment (34 questions), survival (10 questions), and quality of life (49 questions), (Table S1-Table S4). The wording of these questions was deliberately conversational and framed in the first person, mirroring the typical manner in which a patient might pose their queries to the ChatGPT interface.

 Subsequently, the responses generated by ChatGPT were compiled and forwarded to two experts (referred to as MS and EA) to evaluate the accuracy of the information provided. This evaluation process employed a rating system derived from a scoring method established in earlier studies involving ChatGPT.

1. Entirely inaccurate.
2. Partially accurate; includes both correct elements and inaccuracies.
3. Accurate yet non comprehensive; devoid of inaccuracies but lacking in detail that a specialized Gynecologic Oncologist would likely expand upon.
4. Highly accurate and comprehensive; devoid of inaccuracies, covering all essential aspects with no significant additions.

For each question, the initial pair of numeric scores were compared. In cases where these first two scores did not align, the response was forwarded to another expert (TE) for additional evaluation to settle the difference. Should the consensus not be reached on the numeric score by at least two of the experts following the input of the third reviewer, a fourth expert (FE) was consulted. The definitive numeric score for each question was determined by the agreement of at least two experts. It’s important to note that all reviewers were unaware of each other’s assigned scores during the process.^24,25^

 The study analyzed the distribution of ChatGPT response scores both overall and within specific categories of questions. Additionally, it measured the frequency of instances where additional reviewers were necessary to reconcile scoring differences. In a separate analysis, responses were classified into “correct” (scores of 1 and 2) and “incorrect” (scores of 3 and 4). Responses that did not consistently fall into the same category (correct vs. incorrect) between the first two reviewers were removed, and the proportions of scores within the remaining groups were then recalculated. Graph Pad Prism (Version 8.0) was utilized for all statistical analyses.^24^

 This research was not considered to involve Human Subjects, thus it did not require approval from an Institutional Review Board. To ensure the study’s novelty, PubMed searches were conducted before the study began and repeatedly afterwards, using the terms “ChatGPT” in combination with “breast cancer,” “metastatic breast cancer,” and “metastatic breast cancer.”^24^

## Results

**Figure 1:**
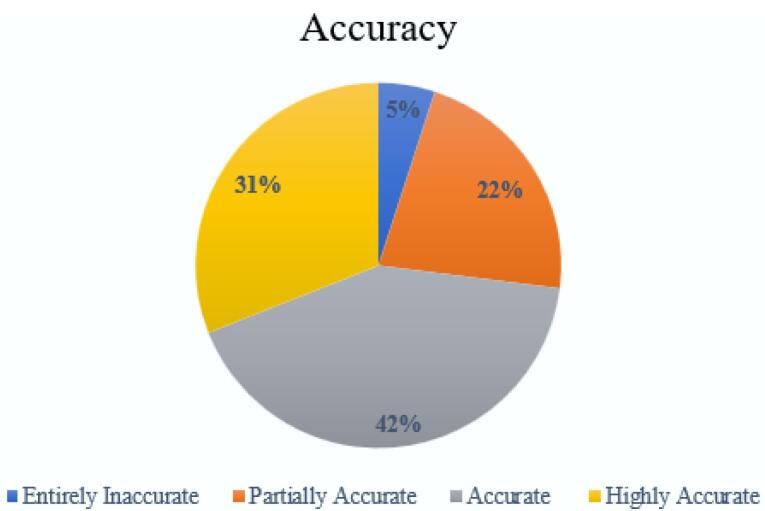
Percentage of different scores for all questions

**Figure 2:**
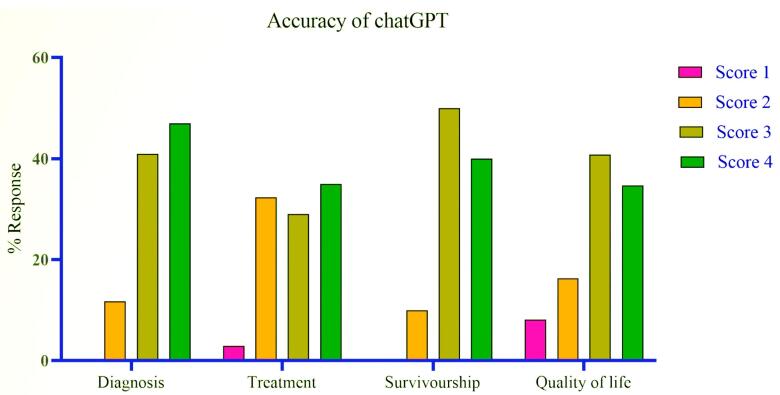
Percentage of scores in each category of questions

 A comprehensive analysis of 100 questions entered into the ChatGPT (Version 4.0) user interface was conducted across four distinct categories: diagnosis, treatment, survival, and quality of life of metastatic breast cancer. These questions were curated to reflect the inquiries commonly presented by patients on reputable cancer information websites and in clinical settings. Two primary experts were tasked with scoring the responses from ChatGPT, utilizing a four-point accuracy scale. In instances of scoring discrepancies, a third and, if necessary, a fourth expert provided additional assessments to reach a consensus. Out of the 100 questions evaluated, 5 responses were categorized as entirely inaccurate. There were 22 responses that were deemed partially accurate, containing some correct elements alongside inaccuracies. The largest number of responses, 42 in total, were considered accurate but not comprehensive, indicating that they contained the right information but lacked detail in certain areas. Finally, 31 responses received the highest accolade of being highly accurate, signifying that they were not only free of inaccuracies but also covered all essential aspects thoroughly. The figures 1 and 2 underscore ChatGPT’s capability to provide reliable information across a spectrum of patient inquiries related to breast cancer. Overall, the proportion of responses that were either accurate but not comprehensive or highly accurate was substantial across all categories, demonstrating a predominant trend towards reliable information provision by ChatGPT.

 In the diagnosis category, none of the 17 ChatGPT responses were categorized as entirely inaccurate. 2 (11.76%) responses were partially accurate, while 7 (41.18%) were accurate but not comprehensive. 8 (47.06%) were rated as highly accurate. The majority responses were rated as highly accurate.

 Within the treatment category, of the 34 responses evaluated, only 1 (2.94%) was found to be entirely inaccurate. Partial accuracy was assigned to 11(32.35%) of responses, and 10 (29.41%) were considered accurate but not comprehensive. Notably, 12 (35.29%) achieved a rating of highly accurate.

 In the survival category, for the 10 responses assessed, none were entirely inaccurate, 1 (10%) were partially accurate, 5 (50%) were accurate but not comprehensive, and 4 (40%) were rated as highly accurate.

 For the quality-of-life category, among 49 responses, 4 (8.16%) were rated entirely inaccurate, 8 (16.33%) were partially accurate, 20 (40.82%) were accurate but not comprehensive, and 17 (34.69%) were deemed highly accurate.

## Discussion

 The analysis of ChatGPT’s responses to breast cancer-related questions reveals a high degree of accuracy, suggesting its potential as a supplementary resource for patient information. Notably, none of the responses in the survival category were entirely inaccurate, and the majority across all categories were at least accurate to some extent. These findings indicate that ChatGPT can generate responses aligned with expert oncological knowledge. However, the presence of responses rated as partially accurate or entirely inaccurate, though a minority, highlights the need for professional oversight when using ChatGPT in a medical context. The study also underscores the complexity of evaluating responses in a nuanced field like oncology. The need for additional expert review in some cases reflects the subjective nature of medical information and the varying levels of detail expected by experts. While ChatGPT can provide immediate responses, which is advantageous in terms of accessibility and time compared to traditional patient-doctor interactions, it cannot replace the personalized advice of healthcare professionals. Moreover, its static knowledge base limits its ability to incorporate the most recent research or clinical guidelines unless periodically updated.

## Conclusion

 In conclusion, ChatGPT demonstrates significant potential as a supplementary tool for patient education and initial information gathering in breast cancer. Its responses generally align with expert knowledge, making it a useful resource when guided and interpreted by healthcare professionals. However, the presence of inaccuracies underscores the need for cautious application without professional oversight. Further research should explore how such AI tools can be integrated into healthcare settings to support patient-provider interactions and enhance health education initiatives. This study contributes to the growing body of literature on AI applications in healthcare and highlights the importance of continuous evaluation and improvement of AI technologies in medical practice.

## Competing Interests

 None to declare.

## Ethical Approval

 Not applicable.

## Supplementary Files

Supplementary File contains Table S1-S4.
