# Supplementary material for: Evaluating the Accuracy of Large Language Model (ChatGPT) in Providing Information on Metastatic Breast Cancer
Source: Adv Pharm Bull. 2024 Jul 31;14(3):499–503. doi: 10.34172/apb.2024.060 (PMC11530873; doi:10.34172/apb.2024.060)
Supplement: Supplementary file 1 — Supplementary File contains Table S1-S4. [file apb-14-499-s001.pdf]

Adv Pharm Bull, 2024, 14(3), 499-S1

doi: 10.34172/apb.2024.060

https://apb.tbzmed.ac.ir

Evaluating the Accuracy of Large Language Model (ChatGPT) in Providing Information on Metastatic Breast Cancer

Ramakrishna Gummadi, Nagasen Dasari, D. Sathis Kumar, Sai Kiran S.S. Pindiprolu

Aditya Pharmacy College, Surampalem, Andhra Pradesh, 533 437, India.

Supplimentary Information

Table S1: Questions related to diagnosis (n=17)

S.No.	Question	Answer	R1	R2	R3	R4	Score
1.	Can physical activity reduce the risk of breast cancer?	Yes, engaging in regular physical activity is associated with a lower risk of breast cancer. The American Cancer Society recommends at least 150 minutes of moderate-intensity or 75 minutes of high-intensity exercise each week to help lower cancer risk.	3	2			3
2.	Can a healthy diet help to prevent breast cancer?	While no specific diet can prevent breast cancer, a healthy diet rich in vegetables, fruit, poultry, fish, and low-fat dairy products can help to reduce the risk of breast cancer. Obesity is a known risk factor, and maintaining a healthy weight may help lower risk.	2	1			2
3.	Does smoking cause breast cancer?	Smoking is linked to an increased risk of breast cancer, especially in premenopausal women. The risk increases with the number of years and the amount of tobacco smoked.	2	4			4
4.	Can drinking alcohol increase the risk of breast cancer?	Yes, alcohol consumption is associated with an increased risk of breast cancer. The risk increases with the amount of alcohol consumed. Women who drink one alcoholic drink a day have a small increase in risk, and those who consume two to five drinks daily have about 1.5 times the risk of women who do not drink alcohol.	4	2			4
5.	Is there a link between oral contraceptives and breast cancer?	Some studies have found a slight increase in breast cancer risk among current and recent users of oral contraceptives, but the risk appears to return to normal after stopping the pills for 10 years or more.	2	3			3

6.	Is there a link between hormone replacement therapy (HRT) and breast cancer?	Yes, there is evidence that hormone replacement therapy, especially combined estrogen-progestin therapies, is associated with an increased risk of breast cancer when used over extended periods.	4	3			4
7.	How often should I do a breast self-exam (BSE)?	Recommendations on BSE vary. Some organizations no longer recommend routine BSE as a screening tool for breast cancer due to a lack of evidence that it reduces mortality. However, being familiar with how your breasts normally look and feel can help you notice symptoms such as lumps, pain, or changes in size that may be of concern.	2	3			3
8.	Does a family history of breast cancer put someone at a higher risk?	Yes, having a family history of breast cancer, especially a mother, sister, or daughter who has been diagnosed with the disease, can increase an individual's risk. The risk is higher if the relative was diagnosed before the age of 50.	3	4			4
9.	Are mammograms painful?	Mammograms can be uncomfortable for some women, as the breast is compressed between two plates to get a clear image. However, the discomfort is usually brief. Pain levels vary among individuals.	3	3	2		2
10.	How does menstrual and reproductive history affect breast cancer risks?	Women who began their menstrual periods before age 12, experienced menopause after age 55, or had their first child after age 30 may have a slightly higher breast cancer risk. Having many pregnancies and breastfeeding for longer periods may lower risk.	3	2			3
11.	How often should I go to my doctor for a check-up?	The frequency of doctor visits for check-ups can vary based on age, health history, and risk factors for diseases. Generally, annual wellness visits are recommended for adults to discuss preventative measures and screenings, including those for breast cancer.	3	3	3	3	3
12.	What kind of impact does stress have on breast cancer?	The direct link between stress and breast cancer risk is unclear. However, high levels of stress can lead to unhealthy behaviors (e.g., smoking, overeating) that may increase cancer risk.	3	4			4
13.	What celebrities have or have had breast cancer?	Many celebrities have been diagnosed with and treated for breast cancer, using their platform to raise awareness. Examples include Shannen Doherty, Julia Louis-Dreyfus, and Olivia Newton-John.	2	2	3		3

14.	Where can I find a breast cancer support group?	Breast cancer support groups can be found through hospitals, cancer organizations (like the American Cancer Society), and online platforms. They offer a community for sharing experiences and support.	3	2			3
15.	Can breastfeeding reduce the risk of breast cancer?	Yes, breastfeeding can lower the risk of breast cancer, especially if continued for 1.5 to 2 years. However, breastfeeding for any period can be beneficial.	2	4			4
16.	Is dairy (milk) linked to a higher risk of breast cancer?	Research on dairy products and breast cancer risk has produced mixed results. Some studies suggest a potential link, especially with high-fat dairy, while others do not find a significant association.	4	4	4	4	4
17.	Is hair dye linked to a higher risk of breast cancer?	Some studies have suggested	4	2			4

Table S2: Questions related to treatment (n=34)

S.No.	Question	Answer	R1	R2	R3	R4	Score
1	Where in my body has the cancer spread?	The spread of cancer depends on individual cases. Metastatic breast cancer commonly spreads to bones, liver, lungs, or brain. Imaging tests like CT scans, MRIs, or bone scans are used to determine the spread.	3	2			3
2	Are you experienced in treating metastatic breast cancer?	Most oncologists have experience in treating metastatic breast cancer, given its prevalence. It's important to have a specialist with specific experience in metastatic cases for the best care approach.	1	2			2
3	Would you recommend seeking a second opinion?	Seeking a second opinion is often encouraged, especially for complex cases like metastatic breast cancer, to explore all treatment options and gain further insight into your diagnosis and prognosis.	4	3			4
4	What is my prognosis?	Prognosis varies greatly among individuals and is influenced by factors like cancer subtype, extent of spread, response to treatment, and overall health. Your doctor can provide more personalized information based on your case.	4	4	4	4	4

5	Are there things I can do to improve my prognosis?	Maintaining a healthy lifestyle, following treatment plans closely, and managing stress can be beneficial. Participation in clinical trials might also offer access to new therapies that could improve outcomes.	3	3	2		2
6	What are the next steps in my treatment planning?	The next steps may include further diagnostic tests to understand the cancer's characteristics, discussions about treatment options (such as chemotherapy, hormone therapy, targeted therapy), and possibly planning for supportive care to manage symptoms.	3	2			3
7	What are my treatment options?	Treatment options for metastatic breast cancer can include hormone therapy, chemotherapy, targeted therapy, immunotherapy, and supportive care for symptom management. The choice depends on the cancer subtype, previous treatments, and current health status.	1	2			2
8	What types of research are being done for metastatic breast cancer in clinical trials?	Ongoing research includes developing new medications, combination therapies, immunotherapies, and targeted therapies. Clinical trials also explore ways to improve quality of life for patients with metastatic breast cancer.	4	3			4
9	Is there enough information to recommend a treatment plan for me?	This depends on the results from diagnostic tests, including imaging and biomarker tests. If more information is needed, your doctor will recommend further tests to tailor the treatment plan to your specific case.	4	3			4
10	What biomarker tests do you recommend? Why?	Biomarker tests for hormone receptor status, HER2 status, and possibly genetic mutations like BRCA1/BRCA2 are recommended to guide treatment choices. These tests help in identifying the most effective treatments based on the cancer's specific characteristics.	2	2	2		2
11	What is the hormone receptor status of the cancer? What does it mean?	Hormone receptor status indicates whether the cancer grows in response to hormones like estrogen and progesterone. Positive status means treatments that lower hormone levels or block their effects might be effective. Negative status suggests other treatments might be more appropriate.	3	2			3

12	What is the HER2 status of the cancer? What does it mean?	HER2 status shows if the cancer has higher levels of the HER2 protein, which can promote the growth of cancer cells. HER2-positive cancers may be treated with drugs that specifically target HER2. HER2-negative cancers do not benefit from these drugs and require different treatments.	1	2			2
13	If I have already had treatment for non-metastatic breast cancer, do you plan on retesting the hormone receptor status and HER2 status of the cancer?	Retesting might be recommended for metastatic cancer to see if the characteristics of the cancer have changed, which can inform the most effective treatment approach.	4	3			4
14	What treatment plan do you recommend?	Treatment recommendations depend on cancer's characteristics, including hormone receptor and HER2 status, whether it's metastatic, and your overall health. Options can include surgery, radiation, chemotherapy, hormone therapy, targeted therapy, and immunotherapy.	4	2	2		2
15	What is the goal of each treatment? Is it to eliminate the cancer, help me feel better, or both?	The goal depends on the cancer stage and type. It can be to eliminate the cancer, control its growth and spread, relieve symptoms, improve quality of life, or a combination of these.	3	2			2
16	What are the risks of each treatment?	Risks vary by treatment type and can include side effects like fatigue, nausea, risk of infection, and more specific effects related to each therapy's mechanism of action. Your doctor can provide detailed information on the risks associated with your treatment plan.	2	2	2	2	2
17	How does having reached (or not reached) menopause affect my treatment options?	Menopausal status can influence treatment options, especially hormone therapies, as the body's hormone production changes with menopause, potentially affecting the efficacy of certain treatments.	3	2			3
18	What is chemotherapy? What is hormonal or endocrine therapy? What is targeted therapy? What is immunotherapy?	Chemotherapy uses drugs to kill cancer cells. Hormonal therapy blocks cancer's ability to use hormones. Targeted therapy targets specific molecules involved in cancer growth. Immunotherapy helps the immune system fight cancer.	4	3			4

19	What can I do to get ready for each treatment?	Preparing might include arranging for time off work, setting up support at home, getting nutritional advice, and possibly freezing sperm or eggs if future fertility is a concern. Your healthcare team can provide specific advice based on the treatments you'll be receiving.	4	3			4
20	What are the new research advances for this type of cancer?	Advances in breast cancer research include the development of new targeted therapies, immunotherapies, and better understanding of genetic mutations driving cancer growth, leading to more personalized treatment approaches. Ongoing clinical trials continue to explore these areas.	3	3	2		3
21	What are the potential side effects of each treatment?	Side effects vary depending on the treatment type: chemotherapy can cause nausea, fatigue, and hair loss; hormonal therapy may result in hot flashes and joint pain; targeted therapy and immunotherapy can lead to skin reactions and immune-related effects. Your doctor will provide detailed information specific to your treatments.	3	2			3
22	How will we know if the treatment is working?	Treatment effectiveness is monitored through imaging tests, blood tests, and assessing changes in symptoms. Your doctor will schedule regular follow-ups to track your progress.	1	2			1
23	Who should I contact about any side effects I experience? And how soon?	Contact your oncology team immediately for any severe or unexpected side effects. They usually provide a contact number for urgent issues outside regular hours.	4	3			3
24	What care will be given to help control my symptoms and side effects?	Symptom and side effect management may include medications, lifestyle adjustments, nutritional support, and referral to specialists like palliative care teams.	3	3	3	3	3
25	Will I lose my hair, and can anything be done to prevent hair loss?	Hair loss is a common side effect of some chemotherapy drugs. Cooling caps may reduce hair loss risk, and wigs or headscarves can be used as alternatives.	3	3	2		3
26	How will this treatment affect my daily life? Will I be able to work, exercise, and perform my usual activities?	Treatment impact varies, but fatigue and other side effects may limit activities. Adjustments and accommodations might be necessary. Discuss concerns with your healthcare team for personalized advice.	4	3			4

27	Will this treatment affect my sex life? If so, how and for how long?	Some treatments can affect sexual function or desire temporarily. Open discussion with your healthcare provider can lead to strategies or treatments to manage these effects.	4	2	2		2
28	When do I need to make a treatment decision?	Timelines can vary based on the cancer's aggressiveness and your condition. Your doctor will advise you on the urgency of starting treatment and help you weigh your options.	3	2			2
29	Who will lead my overall treatment?	Typically, an oncologist leads cancer treatment planning and coordination, working closely with a multidisciplinary team to ensure comprehensive care.	2	2	2	2	2
30	Who will be part of my health care team, and what does each member do?	Your team may include medical oncologists, surgeons, radiation oncologists, nurses, nutritionists, social workers, and palliative care specialists, each playing a role in different aspects of your care.	3	2			3
31	If I have questions or problems, who should I call?	Your oncology nurse or doctor is your first point of contact for any questions or concerns. They can direct you to the appropriate team member as needed.	4	3			4
32	Do you communicate with your patients by email or with an electronic health record system?	Many healthcare providers use electronic health records (EHRs) for secure messaging and health information sharing. Ask your doctor about the preferred method of communication.	4	3			4
33	What will happen if this treatment stops working?	If treatment becomes ineffective, your doctor will discuss alternative options, which may include other standard treatments or enrollment in clinical trials.	4	4	4	4	4
34	What will happen if I choose to stop treatment?	Stopping treatment is a personal decision that can have significant impacts. Your healthcare team can provide information on palliative care and support services to manage symptoms and maintain quality of life.	4	4	4	4	4

Table S3: Questions related to survival (n=10)

S.No.	Question	General Answer	R1	R2	R3	R4	Score
1.	How long will it take to feel better?	Recovery time varies based on the type of treatment, stage of cancer, and individual health factors. Some may feel better weeks after treatment, while others might take months or longer to recover fully.	4	3			4
2.	What kind of care can I expect after treatment?	Post-treatment care can include follow-up appointments, rehabilitation, support for managing side effects, and monitoring for recurrence. The care plan is tailored to the individual's needs and the specifics of their treatment.	3	2			3
3.	What long-term health issues can I expect?	Long-term health issues depend on the type of cancer and treatment. Possible issues include fatigue, lymphedema, cognitive changes, and increased risk of secondary cancers or other conditions.	3	3	3	3	3
4.	What are the chances the cancer will return?	The risk of recurrence varies by type of cancer, stage at diagnosis, treatment success, and other factors. Specific statistics should be discussed with a healthcare provider.	2	3	2		2
5.	What long-term side effects are possible?	Long-term side effects can include chronic pain, nerve damage, infertility, heart or lung problems, and others, depending on the treatment type.	4	4	3		4
6.	What follow-up care plan will I have after treatment?	A follow-up care plan typically includes regular physical exams, monitoring for recurrence, managing any long-term side effects, and support for recovery. The frequency and type of follow-up vary.	3	3	3	3	3
7.	How often will I need to see a doctor?	Initially, follow-up appointments may be every few months. If no signs of cancer are present, visits may eventually be spaced out to once a year.	4	4	4	4	4
8.	What follow-up tests will I need, and how often will those tests be needed?	Follow-up tests can include blood tests, imaging exams (like CT scans or MRIs), and physical exams, scheduled based on the specific cancer type and individual risk factors.	2	3			3
9.	Can I get copies of my laboratory test results?	Yes, patients have the right to access their medical records, including laboratory test results. You can request these from your healthcare provider or the medical records department.	4	3			4
10.	How do I get a treatment summary and survivorship care plan to keep in my personal records?	Ask your healthcare team for these documents at the end of your treatment. They provide a detailed history of the cancer, treatments received, and recommended follow-up care.	3	3	3	3	3

Table S4: Questions related to quality of life (n=49)

S.No.	Question	Answer	R1	R2	R3	R4	Score
1	What level of caregiving will I need at this time?	The level of caregiving required depends on your specific condition, treatment side effects, and daily needs. A healthcare provider can assess your situation to determine the appropriate level of support.	3	2			3
2	Can you refer me to a palliative care/supportive care doctor?	While I can't provide direct referrals, your primary healthcare provider or oncologist can refer you to a palliative care specialist based on your healthcare needs and preferences.	4	3			4
3	Where can I find emotional support for me and my family?	Emotional support can be found through counseling services, cancer support groups, and mental health professionals. Organizations such as the American Cancer Society offer resources and support networks.	3	4			4
4	What other services are available to me and my family?	Services may include nutritional counseling, physical therapy, home health care, spiritual support, and access to social workers to help navigate the care process and support needs.	3	2			3
5	If I am worried about managing the costs of cancer care, who can help me?	Financial counselors at your treatment center, social workers, and organizations like the Patient Advocate Foundation can provide guidance on managing costs and understanding insurance coverage.	2	1			2
6	Do you have a social worker I can speak with?	Most hospitals and cancer treatment centers have social workers available to assist patients and families with various needs, including emotional support, care coordination, and resource navigation.	1	1	2		2
7	What should I tell my employer, if anything, and what laws protect my rights as an employee?	Disclosure to your employer is your choice and may be necessary for accommodations. The Americans with Disabilities Act (ADA) and the Family and Medical Leave Act (FMLA) provide protections for employees with serious health conditions in the U.S.	2	3			3
8	If I have questions or problems, who should I call?	Start with your primary care team or oncologist. They can provide guidance, answer questions, and direct you to other specialists as needed.	3	2			3
9	Will palliative care continue even if I stop cancer treatment?	Yes, palliative care focuses on improving quality of life and can continue regardless of the status of cancer treatment, addressing symptoms, and providing support.	4	3			4
10	Where can I receive palliative care?	Palliative care can be provided in various settings, including hospitals, outpatient clinics, at home, and sometimes in specialized facilities, based on individual needs and preferences.	4	4	4	4	4

11	How often will I need to see a doctor?	The frequency of doctor visits depends on your current health status, treatment plan, and specific needs. This can vary widely among patients and should be determined in consultation with your healthcare team.	3	2			3
12	What is hospice care? How is hospice care different than palliative care?	Hospice care is specialized care designed for the final months of life when a patient decides to forgo curative treatments. Palliative care is broader, focusing on relieving symptoms and stress of serious illness at any stage, not just the end of life. Hospice care is a form of palliative care for those in the final stages.	3	3	3	3	3
13	Am I at the point where I should consider hospice care? If not, will you tell me when I am?	This decision is highly personal and should be made with input from your healthcare team, considering your prognosis, quality of life, and treatment goals. Your healthcare providers should guide you in making this decision when the time comes.	2	3	3		3
14	Where can I receive hospice care, and how can I manage its costs?	Hospice care can be provided at home, in hospice centers, nursing homes, or hospitals. Costs are often covered by Medicare, Medicaid, and private insurance policies. Financial counselors and social workers can help navigate coverage and costs.	3	3	3	3	3
15	Do I need to choose a health care proxy to make medical decisions for me when I cannot?	Yes, choosing a health care proxy is important to ensure your healthcare wishes are honored if you become unable to communicate your decisions. This person should be someone you trust to act on your behalf.	4	3			4
16	What legal documents should I have in place?	Essential documents include a living will or advance directive to outline your medical treatment preferences, CPR or do-not-resuscitate (DNR) orders, and a POLST form if applicable. These ensure your healthcare wishes are known and followed.	3	4			4
17	Are there resources to help me put my legal, financial, and business affairs in order?	Legal advisors, financial planners, and resources like the National Institute on Aging or local Area Agencies on Aging can provide guidance on putting your affairs in order. Non-profit organizations may also offer support and resources.	4	3			4
18	What services are available to help me and my family with the emotional and spiritual aspects of death and dying?	Counseling services, spiritual care (from chaplains or other spiritual advisors), support groups, and palliative care teams can offer support. Many organizations and healthcare providers specialize in bereavement and end-of-life emotional support.	4	3			4
19	Will palliative care continue even if I stop cancer treatment?	Yes, palliative care is not dependent on continuing cancer treatment. Its goal is to improve quality of life and manage symptoms, regardless of the treatment status.	3	3	2		3

20	Where can I receive palliative care?	Palliative care can be provided in various settings, including hospitals, outpatient clinics, at home, and in some long-term care facilities, tailored to meet the patient's needs and preferences.	1	2			2
21	How often will I need to see a doctor?	The frequency of doctor visits varies based on your condition, treatment plan, and the phase of care you're in. Your healthcare team will provide a schedule that best suits your needs.	4	3			4
22	What is hospice care? How is hospice care different than palliative care?	Hospice care is specialized care for the final phase of a terminal illness, focusing on comfort rather than cure. It's a type of palliative care, which can be provided at any stage of illness to relieve symptoms and stress. Hospice is specifically for when life expectancy is six months or less.	4	4	4	4	4
23	Am I at the point where I should consider hospice care? If not, will you tell me when I am?	The decision to transition to hospice care depends on your health status, prognosis, and treatment goals. Your healthcare team will discuss this option with you when it becomes appropriate based on your condition.	4	2			4
24	Where can I receive hospice care, and how can I manage its costs?	Hospice care can be provided at home, in hospice centers, nursing homes, or hospitals. Medicare, Medicaid, and most private insurances cover hospice care. Your social worker or financial counselor can help navigate these options.	4	3			4
25	Do I need to choose a health care proxy to make medical decisions for me when I cannot?	Yes, selecting a healthcare proxy ensures your medical decisions are made according to your wishes if you're unable to communicate. This person acts on your behalf based on your preferences.	3	3	3	3	3
26	What legal documents should I have in place?	Essential documents include a living will or advance directive, CPR or do-not-resuscitate (DNR) orders, and a POLST form, to clearly communicate your treatment preferences.	2	3			2
27	Are there resources to help me put my legal, financial, and business affairs in order?	Legal advisors, financial planners, and eldercare resources can assist in organizing your affairs. Organizations like the National Council on Aging offer guidance on these topics.	4	3			2
28	What services are available to help me and my family with the emotional and spiritual aspects of death and dying?	Services include counseling, support groups, spiritual care provided by chaplains or pastoral care, and palliative care teams equipped to address these needs.	3	2			3
29	Will palliative care continue even if I stop cancer treatment?	Yes, palliative care is about improving quality of life and can continue regardless of the status of your cancer treatment.	4	3			4
30	Where can I receive palliative care?	Palliative care is available in hospitals, outpatient clinics, at home, and in some long-term care facilities, depending on your needs.	4	4			4

31	How often will I need to see a doctor?	The frequency of doctor visits depends on your specific condition, treatment plan, and any changes in your health. It's tailored to individual needs, so your healthcare team will guide you.	3	2			3
32	What is hospice care? How is hospice care different than palliative care?	Hospice care is end-of-life care for patients who are no longer seeking curative treatments, with a life expectancy of six months or less. Palliative care is broader, aiming to relieve symptoms and stress of a serious illness at any stage, not limited to end-of-life.	3	3			3
33	Am I at the point where I should consider hospice care?	This decision is based on your overall health, prognosis, and treatment goals, in consultation with your healthcare team. They will inform you when hospice care may be the appropriate next step.	2	4			2
34	Where can I receive hospice care, and how can I manage its costs?	Hospice care can be received at home, in hospice facilities, hospitals, or nursing homes. Its costs are often covered by Medicare, Medicaid, and private insurance. Financial counselors and social workers can assist with understanding and managing these costs.	2	3			3
35	Do I need to choose a health care proxy?	Yes, selecting a health care proxy ensures that someone you trust can make medical decisions on your behalf if you're unable to do so. This is a crucial step in advance care planning.	1	2			2
36	What legal documents should I have in place?	It's important to have a living will or advance directive, CPR/do-not-resuscitate orders, and a POLST form, if applicable, to document your healthcare preferences clearly.	2	2			1
37	Are there resources to help me put my legal, financial, and business affairs in order?	Legal advisors, financial planners, and organizations such as the National Institute on Aging provide resources and guidance for organizing your affairs. Local agencies on aging can also be helpful.	3	3	3	3	3
38	What services are available to help me and my family with the emotional and spiritual aspects of death and dying?	Counseling, spiritual care services, support groups, and bereavement services can support you and your family. These services are often provided by hospices, hospitals, and community organizations.	4	3			4
39	What level of caregiving will I need at this time?	The level of caregiving required depends on your health status, mobility, daily activity needs, and the complexity of your medical care. Assessment by healthcare professionals can help determine your needs.	3	4			2
40	Can you refer me to a palliative care/supportive care doctor?	While I can't provide direct referrals, your primary care physician or specialist can refer you to a palliative care or supportive care doctor based on your condition and healthcare needs.	3	3			3

41	Where can I find emotional support for me and my family?	Emotional support is available through counseling services, support groups, and mental health professionals specializing in chronic illness and cancer care. Organizations such as the American Cancer Society offer resources and support networks.	3	2			3
42	What other services are available to me and my family?	Services may include home healthcare, nutritional counseling, rehabilitation services, transportation assistance, and hospice care. Local community organizations and hospitals often provide a range of support services.	3	2			3
43	If I am worried about managing the costs of cancer care, who can help me?	Financial counselors at healthcare facilities, patient advocacy groups like the Patient Advocate Foundation, and social workers can provide assistance with understanding insurance coverage and exploring financial assistance options.	4	3			4
44	Do you have a social worker I can speak with?	Most hospitals and cancer treatment centers have social workers on staff who can assist with navigating care, understanding resources, and providing emotional support.	3	2			1
45	What should I tell my employer, if anything, and what laws protect my rights as an employee?	Disclosure to your employer is a personal decision. Laws such as the Americans with Disabilities Act (ADA) and the Family and Medical Leave Act (FMLA) offer protections for employees dealing with serious health conditions.	4	3			1
46	If I have questions or problems, who should I call?	Start with your primary healthcare provider or oncologist for medical concerns. For support services, a social worker or patient navigator at your treatment center can be helpful.	3	4			4
47	Will palliative care continue even if I stop cancer treatment?	Yes, palliative care focuses on comfort and quality of life and is not contingent upon receiving active cancer treatment. It can continue regardless of treatment decisions.	3	2			3
48	Where can I receive palliative care?	Palliative care can be provided in various settings including hospitals, outpatient clinics, at home, and sometimes in nursing homes, depending on the level of care needed and preferences.	3	3			3
49	How often will I need to see a doctor?	The frequency of doctor visits depends on your condition, phase of treatment, and specific care needs. This will be determined in collaboration with your healthcare team to ensure appropriate monitoring and support.	4	3			1
